# Extracorporeal Membrane Oxygenation as a Bridge to Recovery in Children With Severe Necrotizing Pneumonia: Case Report

**DOI:** 10.7759/cureus.85593

**Published:** 2025-06-09

**Authors:** Sajjad M AlKadhem, Nada Aljassim, Nabeel Almashreqi

**Affiliations:** 1 Pediatric Intensive Care Department, King Fahad Medical City, Riyadh, SAU

**Keywords:** ecmo, elso, pediatric, respiratory, severe acute respiratory, vv ecmo

## Abstract

Necrotizing pneumonia is a severe complication of bacterial or viral pneumonia that can lead to refractory respiratory failure in children. Extracorporeal membrane oxygenation (ECMO) has emerged as a vital rescue therapy when conventional treatments fail, primarily serving as a bridge to recovery. We describe the successful use of venovenous ECMO (VV-ECMO) in two pediatric patients diagnosed with severe necrotizing pneumonia.

The first patient, a two-year-old boy, developed necrotizing pneumonia caused by parainfluenza 3 virus. He required seven days of VV-ECMO support for refractory hypoxic hypercapnic respiratory failure. He was decannulated and then extubated on day 14 of his illness to a nasal cannula. The second patient, a five-year-old girl, was diagnosed with necrotizing pneumonia caused by *Streptococcus pneumoniae* and Rhinovirus. She required 30 days of VV-ECMO support. Eventually, she was decannulated and extubated on day 40 of her illness.

VV-ECMO is increasingly used in children with severe pneumonia, including necrotizing forms. Prolonged support, as in the second patient, raises complication risks. CT showed bilateral lung disease with effusions, pneumothoraces, and a possible bronchopleural fistula. Lung rest strategies and minimal ventilator settings - or extubation on ECMO - can aid recovery and manage air leaks. Despite complications like circuit clots and neurologic events, both patients recovered lung function and were discharged. ECMO duration depends on disease severity and patient response.

VV-ECMO can be an effective bridge to treat severe necrotizing pneumonia in children. Early ECMO initiation, lung rest, and targeted antibiotic therapy for active infection are crucial for better outcomes.

## Introduction

Necrotizing pneumonia (NP) is an uncommon but rapidly progressive complication of community-acquired pneumonia in children, marked by parenchymal liquefaction, loss of normal lung architecture, and formation of multiple thin-walled cavities [1]. Although classically attributed to highly virulent bacterial pathogens such as *Streptococcus pneumoniae* and *Staphylococcus aureus*, contemporary series have documented a broader microbiologic spectrum and a rising incidence that parallels increased recognition on high-resolution imaging [2]. Progression to severe acute respiratory distress syndrome (PARDS) is not rare; when refractory hypoxemia or hypercapnia ensues, despite optimized mechanical ventilation, mortality approaches 40%-50%.

Venovenous extracorporeal membrane oxygenation (VV-ECMO) offers time-limited cardiopulmonary support that permits ultra-protective ventilation and provides a time for pulmonary healing. Modern pediatric ECMO cohorts report overall survival near 55%, with respiratory indications - particularly severe pneumonia - constituting the largest subgroup [3]. Observational data from the RESTORE cohort suggest that, in carefully selected children with severe PARDS, ECMO can achieve survival comparable to matched non-ECMO controls while avoiding ventilator-associated morbidity [4]. Nevertheless, specific evidence for VV-ECMO in necrotizing pneumonia remains limited to case reports and small series, and the optimal timing of cannulation is still debated.

## Case presentation

Case 1

A previously healthy, fully vaccinated two-year-old boy, weighing 10 kg, was admitted to the pediatric intensive care unit (PICU) with a three-day history of high-grade fever and progressive respiratory distress. On admission, his vital signs were: temperature 39.5°C, heart rate 160 beats/min, respiratory rate 60 breaths/min, blood pressure 90/50 mmHg, and oxygen saturation 88% on room air. Physical examination revealed tachypnea, intercostal retractions, and decreased breath sounds over the right lung field. Initial chest radiography showed right-sided consolidation with early cavitation, indicative of necrotizing pneumonia. Initial laboratory studies revealed marked leukocytosis and profoundly elevated inflammatory markers (Table 1). Initial management included a high-flow nasal cannula, broad-spectrum antibiotics (vancomycin and meropenem), and antivirals (oseltamivir). Due to worsening respiratory distress and worsening hypoxemia, the patient was intubated. On the third day of PICU admission, he underwent chest tube insertion that drained sterile pus and right-sided thoracoscopic decortication. Next day, the patient required higher settings on conventional mechanical ventilation and was started on high-frequency oscillatory ventilation (HFOV), but the child developed persistent hypoxic hypercapnic respiratory failure and severe pediatric acute respiratory distress syndrome (PARDS). Viral polymerase chain reaction (PCR) revealed parainfluenza 3 virus.

**Table 1:** Case 1 - two-year-old boy with parainfluenza 3 necrotizing pneumonia Interpretation: Initial hyper-inflammatory and hypercapnic profile worsened by day 5, prompting VV-ECMO. All markers subsequently progressed toward normal ranges. VV-ECMO: venovenous extracorporeal membrane oxygenation; WBC: white blood cell count; PaCO₂: partial pressure of carbon dioxide.

Parameter	Reference Range	Day 1	Day 3	Day 5†	Day 10‡	Trend
WBC (×10³ µL⁻¹)	5-15	25 ↑	22 ↑	26 ↑↑	13 (near N)	↓
Platelets (×10³ µL⁻¹)	150-400	320 (N)	290 (N)	210 (N)	240 (N)	↔
C-reactive protein (mg L⁻¹)	<5	180 ↑↑	160 ↑↑	200 ↑↑	50 ↑	↓
Procalcitonin (ng mL⁻¹)	<0.5	10 ↑↑	8 ↑↑	12 ↑↑	1 ↑	↓
Arterial pH	7.35-7.45	7.30 ↓	7.28 ↓	7.05 ↓↓	7.40 (N)	↑
PaCO₂ (mmHg)	35-45	70 ↑	85 ↑↑	105 ↑↑	40 (N)	↓

**Figure 1:**
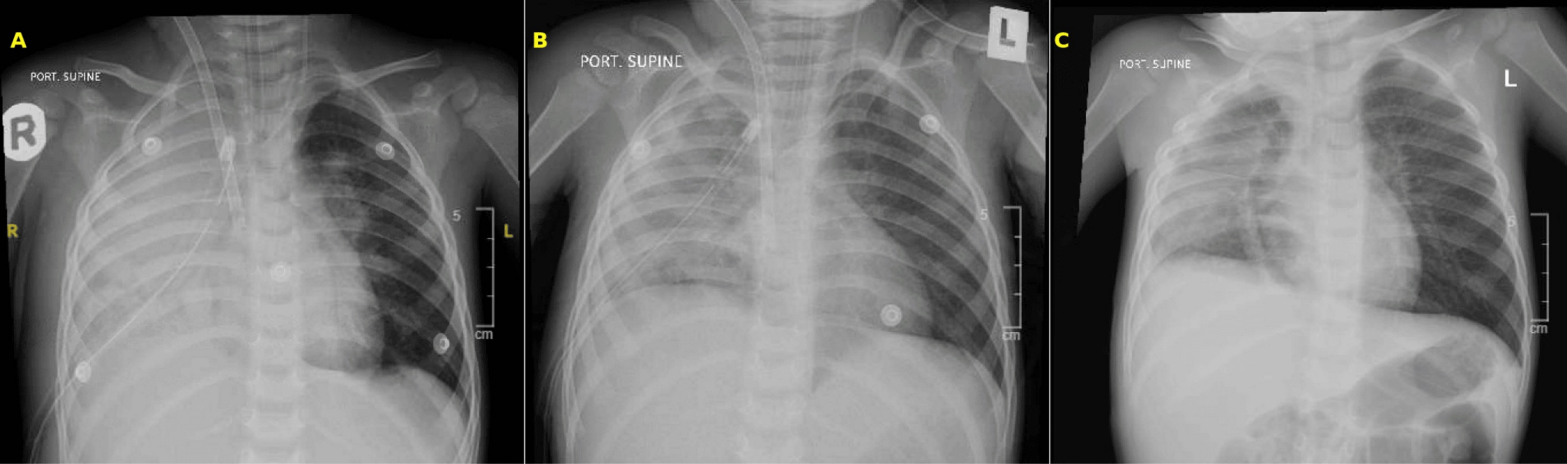
Portable supine anteroposterior chest radiographs from Case 1. (A) Day 0, while on venovenous extracorporeal membrane oxygenation (VV-ECMO): yellow “A” label highlights dense confluent consolidation with small internal lucencies occupying the right middle and lower lobes - early cavitation typical of pediatric necrotizing pneumonia. (B) Day 4 on VV-ECMO: yellow “B” label indicates a pleural drain coursing inferiorly within the now near-homogeneous right-sided opacity and volume-loss pattern, representing postoperative atelectasis and residual pleural fluid. The mediastinum remains mid-line. (C) Day 14 (48 h after ECMO decannulation): yellow “C” label points to coarse linear parenchymal scarring and sub-segmental atelectasis after marked re-aeration of the right lung, indicating resolution of the necrotizing process.

The PICU team consulted the pediatric ECMO team, who decided to initiate VV-ECMO. The patient was cannulated percutaneously via the right internal jugular vein (12 Fr cannula) and right femoral vein (14 Fr cannula). ECMO was initiated with a blood flow rate of 100 mL/kg/min, sweep gas flow of 1-1.2 L/min, and fraction of delivered oxygen (FdO_2_) of 100%. The patient remained on VV-ECMO for seven days, connected to mechanical ventilation on lung-protective ventilation strategy. Following successful weaning from ECMO, the patient was extubated the next day and transitioned to high-flow nasal cannula support for two days. The patient was discharged from the PICU in stable condition to complete the antibiotic regimen. Follow-up chest imaging showed significant improvement in the necrotizing process (Figure 1).

Case 2

A previously healthy five-year-old girl, weighing 18 kg, presented with an upper respiratory tract infection that rapidly progressed to severe respiratory distress. Her condition deteriorated despite non-invasive ventilation attempts, leading to intubation and initiation of conventional mechanical ventilation. The patient developed bilateral pneumothoraces requiring chest tube insertions. A right-sided tension pneumothorax caused bradycardia, necessitating one minute of cardiopulmonary resuscitation. Initial laboratory studies revealed marked leukocytosis and elevated inflammatory markers (Table 2). The patient was diagnosed with necrotizing pneumonia caused by *Streptococcus pneumoniae* and Rhinovirus.

**Table 2:** Case 2 - Five-year-old girl with Streptococcus pneumoniae / Rhinovirus necrotizing pneumonia Interpretation: Inflammatory and gas-exchange derangements peaked early in the ECMO course and showed stepwise improvement, with normalization of most indices by convalescence. ECMO: extracorporeal membrane oxygenation; WBC: white blood cell count; PaCO₂: partial pressure of carbon dioxide.

Parameter	Reference Range	Day 1 (ER)	Day 3	Day 5†	Day 15‡	Day 35§	Trend
WBC (×10³ µL⁻¹)	5-15	18 ↑	24 ↑↑	22 ↑↑	14 ↑	9 (N)	↓
Platelets (×10³ µL⁻¹)	150-400	300 (N)	280 (N)	250 (N)	220 (N)	310 (N)	↔ / mild ↑
C-reactive protein (mg L⁻¹)	<5	150 ↑↑	220 ↑↑	200 ↑↑	80 ↑	25 ↑	↓
Procalcitonin (ng mL⁻¹)	<0.5	7 ↑↑	15 ↑↑	12 ↑↑	3 ↑	0.5 (N)	↓
Arterial pH	7.35–7.45	7.25 ↓	7.20 ↓	7.18 ↓↓	7.35 (N)	7.42 (N)	↑
PaCO₂ (mmHg)	35–45	80 ↑↑	90 ↑↑	92 ↑↑	50 ↑	40 (N)	↓

Initial chest radiographs revealed bilateral diffuse opacities consistent with severe pneumonia. The patient developed refractory hypercarbic respiratory failure secondary to necrotizing pneumonia, requiring ECMO support. Cannulation was performed percutaneously, with a 17 Fr drainage cannula inserted into the femoral vein and a 15 Fr return cannula into the internal jugular vein. Initial ECMO flow was set at 80 mL/kg/min.

Chest computed tomography (CT) scan revealed bilateral loculated pleural effusions, bilateral pneumothoraces (hydropneumothorax), complete bilateral lung collapse, left lung abscess, fluid-filled bronchi bilaterally, and high suspicion of bronchopleural fistula in the right upper bronchus.

The ECMO course was challenging, with drainage insufficiency requiring troubleshooting and the addition of a larger drainage cannula. A circuit exchange was necessary by the seventh day of ECMO, which was complicated by vasodilation and hemodynamic instability. Later, brain CT imaging revealed multifocal supratentorial acute hemorrhagic foci surrounded by cytotoxic edema, which improved in follow-up images. The patient experienced subclinical seizures that were controlled with anti-seizure medication (ASM) (levetiracetam and phenobarbital).

**Figure 2:**
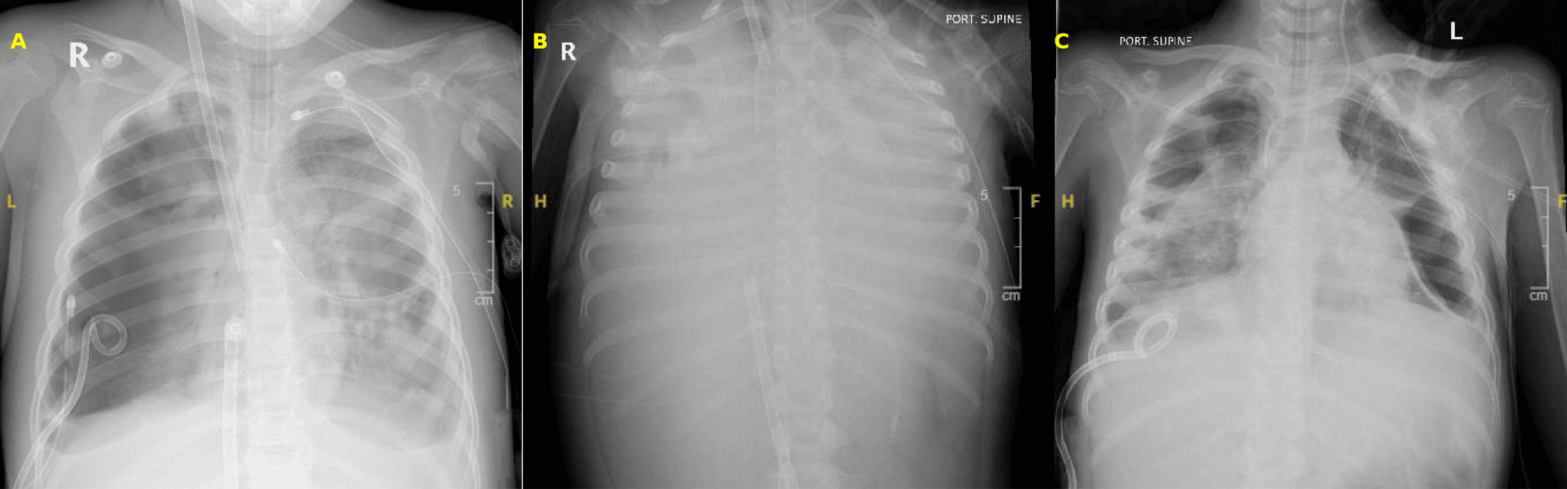
Portable supine anteroposterior chest radiographs from Case 2. (A) Day 0 on VV-ECMO: yellow “A” label marks bilateral chest tubes placed for pneumothoraces amid patchy alveolar opacities consistent with early severe pneumonia complicated by barotrauma. (B) Day 5 on VV-ECMO: yellow “B” label emphasizes “white-out” of both lungs with air–fluid levels - severe acute respiratory distress syndrome under extracorporeal support. Endotracheal tube and pleural drains are visible. (C) Day 32 (48 h post-decannulation): yellow “C” label denotes a persistent left pleural drain and residual perihilar/basal streaky opacities, compatible with a resolving bronchopleural fistula after re-expansion of both lungs.

Serial X-rays revealed organized collection in the right lung, which was discussed for possible surgical intervention. This was deferred with consideration for future video-assisted thoracoscopic surgery (VATS) and decortication (Figure 2).

By day 20 of ECMO, the patient showed improved lung compliance and chest expansion, with tidal volumes increasing to 4.5 mL/kg. Throughout the ECMO run, intermittent air bubbling from both chest tubes persisted.

After 30 days of VV-ECMO support, the patient was successfully decannulated. She was extubated to a high-flow nasal cannula and gradually weaned to a conventional nasal cannula over three days. Post-discharge from the PICU, the patient underwent rehabilitation for critical-illness polyneuromyopathy and followed up with pulmonology for two weeks until the air leak improved.

## Discussion

Necrotizing pneumonia in children represents the severe end of the community-acquired pneumonia spectrum, where exuberant inflammation, microvascular thrombosis, and parenchymal liquefaction culminate in extensive cavitation and, frequently, pleural space complications [1, 2], PARDS is common and portends a mortality approaching 40% when conventional lung-protective ventilation fails [4]. In this context, VV-ECMO serves as a rescue strategy that off-loads the injured lung and permits ultra-low tidal-volume ventilation while the underlying infection and inflammatory cascade are treated.

The Second Pediatric Acute Lung Injury Consensus Conference (PALICC-2) recommends considering ECMO when oxygenation index (OI)>40 or PaO₂/FiO₂<60 persists for >6 h despite optimized ventilator strategies, inhaled pulmonary vasodilators, and prone positioning [5]. Both patients in our series met these criteria, with escalating hypercapnic acidosis despite high-frequency oscillatory ventilation. Early cannulation - within 24 h of meeting PALICC thresholds - has been associated with reduced ventilator-induced injury and fewer extra-pulmonary organ failures [3,4], and was pursued in Case 1. In contrast, the protracted course preceding cannulation in Case 2 illustrates how delayed initiation can translate into longer ECMO runs and higher complication rates.

Both children were supported with dual-site VV-ECMO, reflecting current pediatric practice patterns that favor venovenous support for isolated respiratory failure [3]. Adequate drainage was particularly challenging in Case 2, necessitating the insertion of a second drainage cannula and eventual transition to an adult-size circuit interventions that underscore the importance of frequent circuit performance assessment and the readiness to escalate hardware when resistance or negative-pressure alarms jeopardize flow.

Balancing thrombotic and hemorrhagic risks remains a cornerstone of ECMO care. Traditional unfractionated heparin was complicated by fluctuating anti-Xa levels in Case 2, prompting a switch to a bivalirudin-based protocol that paralleled emerging pediatric data demonstrating lower bleeding rates and equivalent (if not superior) survival with direct thrombin inhibition [6]. Ongoing trials aim to clarify optimal monitoring targets and cost effectiveness of this strategy in children.

Intracranial hemorrhage and electrographic seizures complicated the prolonged run in Case 2. A 2022 systematic review reported a pooled seizure incidence of 15% in pediatric ECMO, with subclinical events comprising three-quarters of cases and doubling short-term mortality [7]. Continuous EEG and cranial imaging were pivotal in our patient, allowing rapid antiepileptic therapy and anticoagulation titration that likely limited secondary injury.

Survival alone is an incomplete success metric. Up to one-third of pediatric ECMO survivors exhibit new functional morbidity at hospital discharge, and physical, cognitive, or psychosocial impairments may emerge months later [8]. Our second patient demonstrated critical-illness polyneuromyopathy and behavioral withdrawal, necessitating a multidisciplinary rehabilitation program. Early mobilization, structured sedation-weaning protocols, and longitudinal follow-up clinics are increasingly recognized as essential to optimize the quality of survival.

## Conclusions

This dual case report illustrates the successful use of VV-ECMO in managing severe necrotizing pneumonia in young children while also highlighting the potential for varied clinical courses and outcomes. It underscores the importance of early recognition of deteriorating respiratory status, timely initiation of extracorporeal support, and a comprehensive, multidisciplinary approach to patient care.

The significant variability in clinical course and potential for complications emphasize the need for careful patient selection, individualized management strategies, and comprehensive follow-up care. Further research is needed to refine patient selection criteria, optimize ECMO management strategies, and improve long-term outcomes in this challenging patient population.
